# Left atrial appendage closure versus anticoagulation in the management of atrial fibrillation: a systematic review, meta-analysis, and meta-regression analysis

**DOI:** 10.1097/MS9.0000000000003703

**Published:** 2025-08-12

**Authors:** Gurmehar Singh, Hyma Bamba, Chiranjeevee R Saravanan, Arvind Dinesh, Sai Harini Chandrasekaran, Jobby John, Pugazhendi Inban, Omniat Amir Hussin

**Affiliations:** a Internal Medicine, Government Medical College and Hospital, Chandigarh, India; b Internal Medicine, Madras Medical College, Chennai, India; c Internal Medicine, Kempegowda Institute of Medical Sciences, Bangalore, India; d Internal Medicine, Vijaya Hospitals, Chennai, India; e Cardiology, Dr. Somervell Memorial CSI Medical College and Hospital Karakonam, Trivandrum, India; f Internal Medicine, St. Mary’s General Hospital and Saint Clare’s Health, Denville, NY, USA; g Internal Medicine, Manhal University, Khartoum, Sudan

**Keywords:** anticoagulation, atrial fibrillation, direct oral anticoagulants, left atrial appendage closure, non-valvular atrial fibrillation, warfarin

## Abstract

**Background:**

Left atrial appendage closure (LACC) is a nonpharmacological option for individuals with atrial fibrillation (AF). LAAC procedure is characterized by its minimally invasive nature and is considered an alternative to anticoagulants that were usually the first line of treatment for patients at risk for blood clotting.

**Methods:**

A literature search of three databases was conducted and relevant randomized controlled trials (RCTs) were selected. A meta-analysis was performed using a random effect model to calculate risk ratios with 95% confidence intervals, and only the articles with the longest follow-up were included in the meta-regression analysis. Bias was assessed using standard methodologies. We included RCTs involving adult patients with AF that compared LAAC with oral anticoagulation therapy. Non-randomized studies, case series, animal studies, and non-English publications were excluded.

**Results:**

We included three studies with a 1516 sample size. All three trials investigated the composite hemorrhagic stroke, all-cause mortality, and non-procedural major bleeding as primary outcomes in addition to the risk of systematic embolism, ischemic stroke, and all major bleeding. Compared to anticoagulation medical therapy, the LAAC device showed a statistically significant reduction in the risk of the composite primary outcome, cardiovascular (CV) death, all-cause deaths, and nonprocedural bleed. When compared to medical management, there was no statistically significant difference in the risk of systemic embolism and all-stroke in the LAAC device group. The age had no significant impact on the primary efficacy outcome using the Meta-regression analysis.

**Conclusion:**

LAAC has comparable efficacy in preventing strokes. The risk of CV death, all-cause deaths, and nonprocedural relevant bleeding, all of which are associated with the composite primary outcome is lower in the LAAC group versus the oral anticoagulation therapy and can have even greater efficacy with a longer follow-up period.

## Introduction

Irregular heartbeats, known as arrhythmia, present as a disturbance in the heart’s rhythm. A prevalent form of this is atrial fibrillation (AF). AF is recognized as a global health concern on the rise, across the world^[1]^, whose burden is expected to increase by >60% in the next 10 years^[2]^. Importantly, its significance is characterized by its contribution to morbidity and mortality risks^[3]^ in addition to blood clot formation that can lead to stroke and heart failure.

AF significantly raises the risk of stroke increasing it by five times compared to those with a normal heart rhythm^[4]^. This higher risk is attributed to the development of blood clots in the “left atrial appendage (LAA).” When these clots break free and journey to the brain, they can obstruct blood vessels resulting in a stroke^[5]^. While oral anticoagulation therapy (OAC) is currently the approach for preventing strokes in AF patients its effectiveness is hindered by factors like adherence to medication chances of bleeding and potential interactions with other drugs^[6]^. These constraints highlight the need to explore treatments such, as left atrial appendage closure (LAAC), which seeks to obstruct the LAA and prevent clot formation entirely^[7]^. There are many management and prevention options to control AF and its complications such as blood thinners (anticoagulants – OAC – vitamin K and non-vitamin K antagonists (VKAs) [NOAC]), antiarrhythmic medications, and non-invasive procedures such as LAAC as an alternative option^[8]^.

LAAC is a type of surgery that’s less invasive and aims to seal off the atrial appendage (LAA) a small pouch, in the left atrium wall. By either removing or closing this sac, the risk of stroke or blood clot formation can be minimized. As an alternative to anticoagulants, in populations that cannot receive OAC or to avoid the complications of medical drugs, LAAC devices have been developed and achieved promising results in stroke or CV death prevention, LAAC traps the blood clot before it exits^[9]^. There are available devices of LAAC with significant sealing properties, such as Watchman and Amplatzer. According to Gloekler *et al*^[8]^, Amplatzer revealed similar safety but superior efficacy and a reduction in all-cause and cardiovascular mortality, and it has a net clinical benefit that makes it an attractive alternative to medical therapy. Aonuma *et al*^[10]^ confirmed that WATCHMAN is a safe and effective alternative therapy in the Japanese population for stroke risk reduction in the SALUTE trial.

A growing body of literature has been investigating the clinical outcomes associated with LAAC compared to OAC in the AF population. According to Nielsen *et al*^[11]^, in a comparison between LAA occlusion (LAAO) with DOACs in high-risk AF populations, they found that in terms of efficacy, both have the same stroke prevention but lower risk of mortality and bleeding. To thoroughly evaluate the effectiveness and safety of LAAC compared to OAC for preventing stroke in AF, we conducted this systematic review that includes three key clinical trials: the PROTECT AF trial^[12]^, the PREVAIL trial^[13]^, and the PRAGUE-17 trial^[14]^. These research studies are considered a cornerstone in presenting evidence regarding the use of LAAC for preventing strokes in individuals with AF. Offering insights into outcomes such as the risk of stroke bleeding complications and overall safety profiles.

Importantly, while systematic reviews typically focus on published and completed trials, this particular review combines data from ongoing studies like “LAACS-2 study”^[7]^ and “OPTION”^[15]^ with well-established trials such as PROTECT AF, PREVAIL, and PRAGUE 17 besides their long-term outcomes. By including these studies, we can stay updated on the advancements in the field of LAAC for stroke prevention in AF. Even though the final results of these studies may not be finalized, yet initial findings and participant characteristics can provide insights into the potential effectiveness and safety of LAAC. This information can help shape practices and guide future research endeavors.

## Methods

“Preferred Reporting Items for Systematic Review and Meta-Analysis (PRISMA)” was followed to conduct this systematic review^[16]^, Complying with the methodological best practices for systematic reviews as outlined in the Cochrane Handbook^[17]^.

### Criteria of the included studies

- Population: Studies on Patients with AF.
- Intervention/exposure: Studies that used left atrial appendage closure.
- Comparator: Studies that used medical anticoagulation.
- Outcome: Studies investigated the composite hemorrhagic stroke, all-cause mortality, and non-procedural major bleeding as a primary outcome in addition to the risk of systematic embolism, ischemic stroke, all major bleeding, and all stroke.
- Study design: Randomized controlled trials (RCT).

We excluded articles with the following characteristics:

- Observational studies, case reports, conference abstracts, case series, thesis.
- Animal studies were excluded.
- Studies where the patients were not diagnosed with hypertension.
- Studies not in the English language.

**HIGHLIGHTS**

- Compared to anticoagulation, left atrial appendage closure (LAAC) demonstrates superior efficacy in reducing composite primary outcomes, cardiovascular (CV) death, and all-cause mortality. Meta-analysis results reveal a pooled effect size of 0.75 (95% CI [0.57, 1.00], *P* = 0.05), with homogeneity across pooled studies (Chi^2^ = 3.05, *P* = 0.22; *I*^2^ = 34%).
- All-cause death risk is significantly lower with LAAC compared to anticoagulation, as evidenced by a pooled effect size of 0.77 (95% CI [0.62, 0.96], *P* = 0.02), with minimal heterogeneity (Chi^2^ = 0.04, *P* = 0.84; *I*^2^ = 0%).
- While LAAC demonstrates favorable trends in reducing all strokes and systemic embolisms compared to anticoagulation, statistical significance is not achieved. However, LAAC significantly reduces the risk of CV deaths with a pooled effect size of 0.64 (95% CI [0.45, 0.90], *P* = 0.010), and nonprocedural relevant bleeding with a pooled effect size of 0.52 (95% CI [0.39, 0.70], *P* < 0.0001).
- Meta-regression analysis indicates age has no significant impact on LAAC’s primary efficacy outcome (*P*-value = 0.107), strengthening the robustness of LAAC’s effectiveness across different age groups.

### Database search

**Figure 1.**
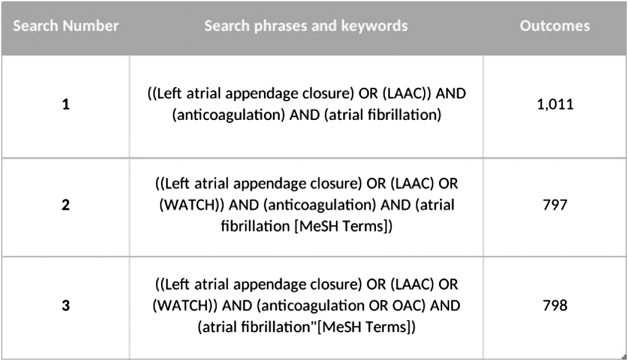
Database search strategy.

We performed database search on Scopus, PubMed, and Web of Science, for relevant studies. We used MESH Keywords for an accurate search strategy on the PubMed database: ((Left atrial appendage closure) OR (LAAC) OR (WATCH)) AND (anticoagulation OR OAC) AND (atrial fibrillation”[MeSH Terms]) (Fig. 1).

### Screening and inclusion criteria

The results of the database research were semi-automatically screened using Rayyan^[18]^. The studies were screened in two stages. Initially, titles and abstracts of potential clinical studies were screened. In the second phase of the process regarding further eligibility screening, full-text articles were retrieved from selected abstracts. Literature research and the screening process had been done independently by three review co-authors, any differences were settled through discussion or, by involving another reviewer.

### Data extraction and analysis

The obtained data were separated into a uniform data retrieval sheet for all included studies. To provide a comprehensive understanding of the data included in those studies, data were extracted in four major categories: study characteristics, study population characteristics, study outcomes, and risk of bias (ROB) domains. In addition to the ID, country, and date of publication. Statistical calculations were conducted using a random effect model with inverse variance weighting to determine risk ratios (RRs) along with 95% confidence intervals (CIs) using Meta-analysis software (RevMan 5.4.1).

### Risk of bias assessment

In our review, we assessed the bias in the studies using the Cochrane ROB tool. The Cochrane ROB tool evaluates bias across seven domains; sequence generation, allocation concealment, blinding of investigators and patients blinding of outcome assessors incomplete outcome data, selective outcome reporting, and other sources of bias. After an analysis of the data provided, each study was categorized as “risk,” “high risk,” or “unclear” based on the level of bias identified.

### Publication bias

The publication bias does not apply to this systematic review in respect of Egger *et al*^[19]^, because the number of studies that have been included in this review was less than ten.

### Quality evaluation

**Figure 2.**
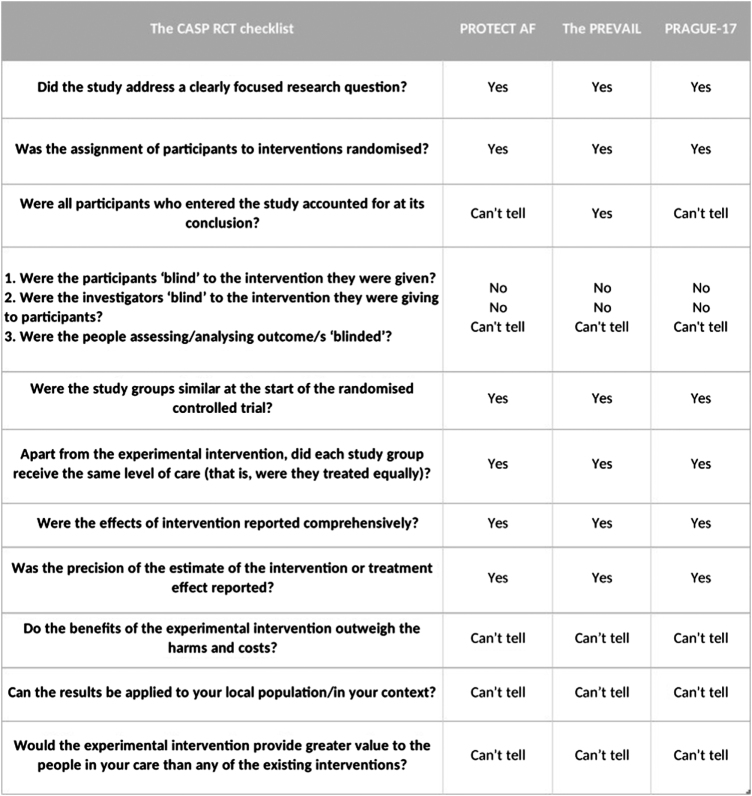
Quality evaluation.

The following 11 questions of the CASP Randomized Controlled Trial Standard Checklist have been used to assess the trustworthiness of the included studies and to achieve the optimal Impact value in healthcare decisions, categorized into three sections evaluating whether the basic study design is valid for an RCT, how the study methodologically sound, and assessing the results (Fig. 2).

### Data synthesis and heterogeneity

In the analysis of three RCTs, the main focus was, on the effectiveness in preventing stroke systemic embolism (SE) and CV death. This analysis involved using Meta-analysis software (RevMan 5.4.1) to assess outcomes such as all strokes, systemic embolisms (SEs), CV deaths, all-cause deaths, and nonprocedural relevant bleeding. Statistical calculations were conducted using a random effect model with inverse variance weighting to determine RRs along with 95% CIs. The heterogeneity among the studies was evaluated through tests like the Cochran *Q* test (test) and Higgins and Thompson *I* squared test. If the *P*-value from the test is below 0.1 and the *I*^2^ value is above 50%, it indicates significant heterogeneity, within the data set^[20]^. Meta-regression analysis will be included to evaluate the demographic characteristic (age) impact on the primary efficacy outcome using OpenMeta[Analyst].

## Results

### Literature search results

**Figure 3.**
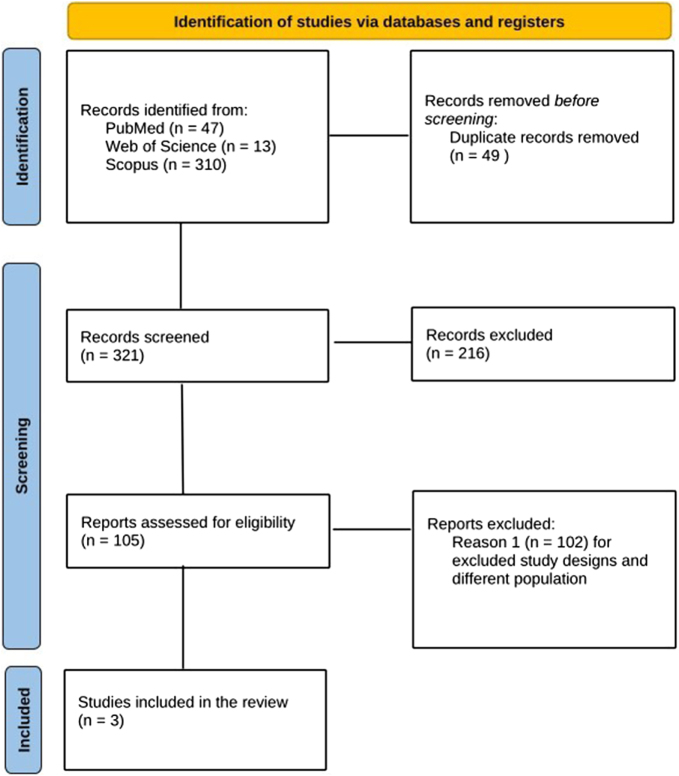
PRISMA flow diagram.

From the literature research, 358 records were obtained. Of them, 49 were identified by the Rayyan intelligent tool as duplicates. After filtering out reviews and summaries, we examined 105 articles, in detail. Among them, we found three articles that reported RCTs for inclusion in our review and meta-analysis. The PRISMA flow diagram is illustrated in Fig. 3.

### Characteristics of the studies included

**Figure 4.**
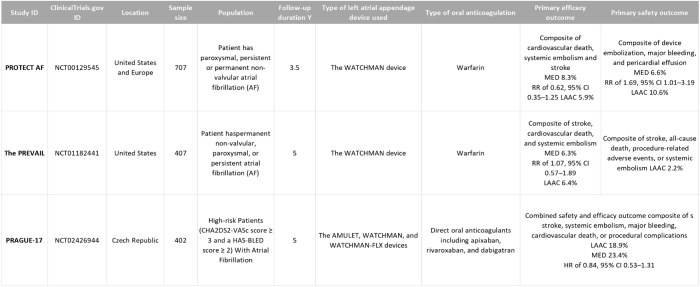
Summary of the included studies results.

**Figure 5.**
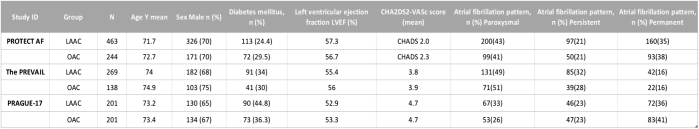
Characteristics of the population in the included studies.

All studies were RCT studies where patients with AF were involved randomly to receive LAAC closure devices or anticoagulation medical therapy. Studies typically included adults with AF. Two studies (PROTECT AF and The PREVAIL) took place in the United States but PRAGUE-17 took place in the Czech Republic. All trials are registered on clinicaltrials.gov from older to newer, respectively (NCT00129545, NCT01182441, NCT02426944). All three RCTs investigated the composite hemorrhagic stroke, all-cause mortality, and non-procedural major bleeding as a primary outcome in addition to the risk of systematic embolism, ischemic stroke, all major bleeding, and all stroke. The characteristics of the participants in the included studies are summarized and collected in Figures 4 and 5, respectively.

### Risk of bias evaluation

**Figure 6.**
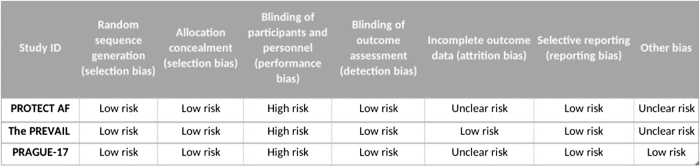
Risk of bias assessment.

**Figure 7.**
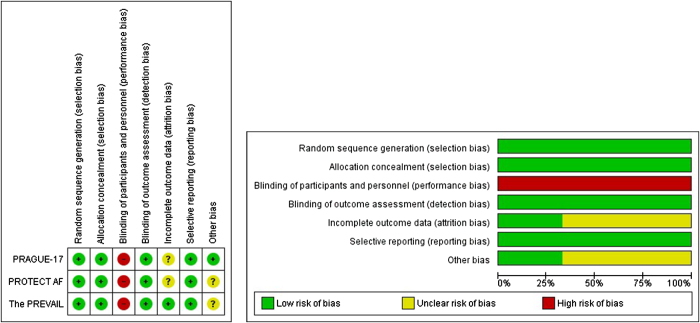
Risk of bias graph and summary.

According to the evaluation of bias, the studies included varied, in quality from low, to high. All of the summary of the assessment of bias, ROB graph and summary are shown in Figures 6 and 7, respectively. Blinding was a concern in the three included studies, but the different nature of the intervention makes it acceptable, otherwise, for every co-primary outcome assessed, all RCTs were classified as low risk. Several studies have limitations that affect how widely their findings can be applied. To start with the PREVAIL and PROTECT AF trials only included suitable patients, for long-term anticoagulation. Additionally, neither study compared LAA closure to NOACs, an alternative for preventing strokes. Furthermore, we still don’t know the long-term impact of LAAO on the heart’s function. Lastly, PRAGUE 17 had limited power to analyze aspects of the combined endpoint (stroke vs. Bleeding). Although the high-risk population in PRAGUE 17 provided power for the endpoint longer follow-up is necessary to evaluate any lasting differences.

### Primary outcomes

#### Primary efficacy: stroke/CV death/SE

The overall RR between the LAAC device group and the anticoagulation group favored LAAC with pooled effect size (0.75, 95% CI [0.57, 1.00], *P* = 0.05), and pooled studies were homogenous (Chi^2^ = 3.05, *P* = 0.22; *I*^2^ = 34%).

#### All-cause death

The overall RR between the LAAC device group and the anticoagulation group favored LAAC with pooled effect size (0.77, 95% CI [0.62, 0.96], *P* = 0.02), and pooled studies were homogenous (Chi^2^ = 0.04, *P* = 0.84; *I*^2^ = 0%).

#### All strokes

The overall RR between the LAAC device group and the anticoagulation group favored LAAC with pooled effect size (0.91, 95% CI [0.61, 1.35], *P* = 0.65), and pooled studies were homogenous (Chi^2^ = 1.99, *P* = 0.37; *I*^2^ = 0%).

#### Systemic embolisms

The overall RR between the LAAC device group and the anticoagulation group favored LAAC with pooled effect size (1.27, 95% CI [0.21, 7.67], *P* = 0.79), and pooled studies were homogenous (Chi^2^ = 1.18, *P* = 0.55; *I*^2^ = 0%).

#### Cardiovascular deaths

The overall RR between the LAAC device group and the anticoagulation group favored LAAC with pooled effect size (0.64, 95% CI [0.45, 0.90], *P* = 0.010), and pooled studies were homogenous (Chi^2^ = 0.05, *P* = 0.83; *I*^2^ = 0%).

#### Nonprocedural relevant bleeding

The overall RR between the LAAC device group and the anticoagulation group favored LAAC with pooled effect size (0.52, 95% CI [0.39, 0.70], *P* < 0.0001), and pooled studies were homogenous (Chi^2^ = 0.26, *P* = 0.61; *I*^2^ = 0%).

**Figure 8.**
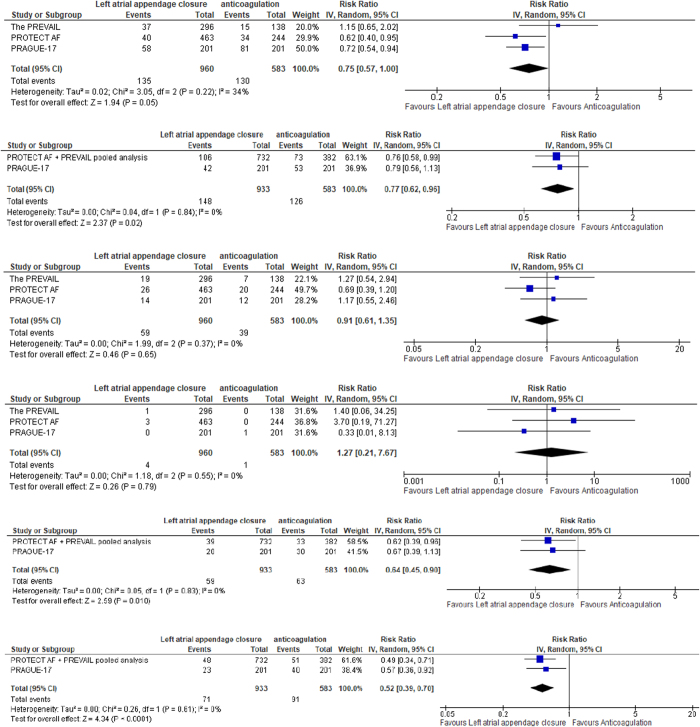
Forest plot of the included studies.

Therefore, compared to the anticoagulation medical therapy, the LAAC device showed a statistically significantly lower risk of the composite primary outcome, CV death, all-cause deaths, and nonprocedural relevant bleeding. When compared to medical management, the research findings did not show any variance in the embolism risk and all types of strokes, within the LAAC device group. The results and the forest plot of each outcome are displayed respectively in Figure 8.

#### Meta-regression analysis

**Figure 9.**
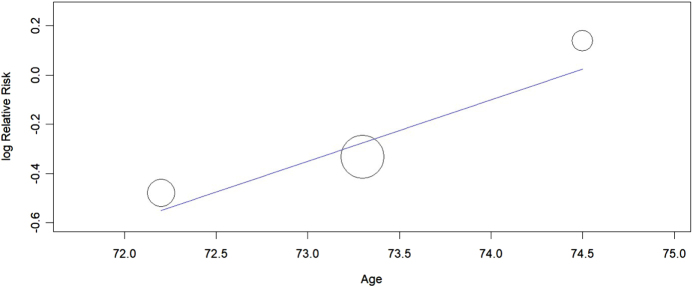
Meta-regression plot.

According to the data retrieved from the long-term outcomes reported from PRAGUE-17^[21]^, the PREVAIL and PROTECT AF Trials^[22]^, the age has no significant impact on the primary efficacy outcome with a *P*-value of (0.107), the regression graph is shown in Figure 9. Due to the limited number of studies (*n* = 3), sensitivity analyses were not conducted as they would lack sufficient power to yield robust conclusions.

## Discussion

The most common arrhythmia in the world is non-valvular AF, which can reveal itself as a stroke as a first symptom in asymptomatic patients^[23]^. Many studies were interested in studying variable methods to manage patients with AF from further life-threatening complications, last few years, LAAC proved its safety and efficacy which were studied by many but fewer RCTs. Methodological analysis to overcome the heterogeneity between studies that evaluated LAAC versus OAC, only RCTs included in this systematic review and meta-analysis, the main findings of PROTECT AF, The PREVAIL, and PRAGUE-17 are that LAAC showed a comparable efficacy compared to OAC in AF patients in terms of stroke in addition to a decrease in risk of all-cause death, CV death, and major relevant bleeding.

The long-term data gathered from the PRAGUE 17 trial^[21]^ continue to support the use of LAAC as an option compared to direct oral anticoagulants (DOACs) in preventing significant CV, neurological, and bleeding events, among high-risk patients with AF. The decrease in procedural bleeding incidents associated with LAAC in the study is worth noting. Similarly, when looking at the combined outcomes from the PREVAIL and PROTECT AF trials^[22]^ it is evident that the Watchman LAAC device provides stroke prevention in valvular AF patients on par with warfarin therapy. One advantage of LAAC lies in its ability to reduce bleeding events, and hemorrhagic strokes potentially leading to lower rates of disability and mortality compared to warfarin treatment.

Many observational studies discussed this hypothesis, the most recent one is Zeitler *et al* (2023)^[24]^ which mainly aims to compare the effectiveness of LAAC versus OAC in terms of sex. They reported that LAAC was associated with a noticeable reduction in the risk of stroke, long-term bleeding, and death in both men and women. According to *Falasconi* et al. (2023)^[25]^, in the Italian population within a reasonable follow-up period of 3.4 years, reported that within the LAAC group, no major procedural bleeding occurred in addition to a lower risk rate in cardiac embolism, they supported the current evidence by reporting that LAAC is an effective management option in non-valvular AF population who exposed to cardiac embolism in despite of the current OAC therapy. In terms of Ding *et al* (2022)^[26]^, 108 697 participants were involved and indicated that LAAC could be a potential option instead of non-vitamin K antagonist oral anticoagulants (NOACs), for preventing strokes in individuals, with AF.

The most recent ongoing clinical trials addressing the same research question are Wazni *et al* 2022^[15]^ (OPTION; NCT03795298) and Madsen *et al* (2023)^[7]^ (trial registration: NCT03724318). Regarding the OPTION trial, it was conducted considering the current guidelines that recommend that patients should continue taking anticoagulants (OAC) after undergoing AF ablation due, to uncertainties regarding the long-term maintenance of normal heart rhythm and left atrial function. However, this approach presents challenges in terms of compliance as studies have shown that up to 23% of individuals stop taking OAC medications after the ablation procedure according to the ORBIT AF registries^[27]^. Additionally, there is a concern about an increased risk of bleeding associated with continuing OAC therapy despite its benefits in preventing strokes and reducing the risk of hemorrhage. Their hypothesis suggests a potential solution to address these challenges is through LAA closure using devices like WATCHMAN FLX, which could offer an alternative for stroke prevention regardless of the outcome of the ablation procedure. This approach aims to lower stroke risk by preventing blood clots from forming in the LAA and potentially avoiding bleeding complications linked with OAC therapy. The strengths of the OPTION trial lie in being the first randomized controlled study that compares LAA closure using WATCHMAN FLX with OAC therapy following AF ablation. This trial fills a gap in existing evidence compared to studies, on WATCHMAN devices that primarily focused on warfarin use. Regarding Madsen *et al* (2023), the researchers are looking into whether sealing off the appendage (LAA) during open heart surgery can lower the chances of having a stroke compared to the usual practice of keeping the LAA unsealed. While the latest recommendations suggest that surgical closure of the appendage (LAA) may be beneficial, for certain patients with AF although there is not enough evidence to support its widespread application, This RCT’s goal is to investigate whether LAA closure decreases the risk of stroke or transient attack in individuals undergoing surgery and to assess the effectiveness of this strategy in patients both with and without AF taking into account stroke risk scores (such, as CHA2DS2 VASc). Recent developments in LAAC technology, including the WATCHMAN FLX and Amulet devices, have shown improved efficacy and safety profiles. Long-term data from trials such as OPTION and LAACS-2 are expected to provide further clarity on the comparative effectiveness of LAAC versus anticoagulation, particularly in post-ablation settings and during concomitant cardiac surgery.

## Limitations

The included studies used types of anticoagulation methods (VKA, DOAC, or both), and varying definitions of bleeding across the studies make it difficult to clearly understand how treatments affect patient groups or their risks of bleeding. Using procedural anticoagulation, regimens involving VKAs followed by dual or single antiplatelet therapy could introduce bias, especially in estimating bleeding risks. Moreover, the absence of discharge plans and potential differences in device types (Watchman vs. Amplatzer) might impact the results.

## Conclusion

LAACs have comparable effectiveness to DOACs/warfarin for stroke prevention in high-risk AF patients based on PRAGUE 17, PREVAIL & PROTECT AF, Reduced risk of bleeding incidents with LAAC when compared to warfarin as shown by PRAGUE 17 and PROTECT AF studies, and potential for decreased disability and mortality rates associated with LAAC due to a reduction, in bleeding incidents according to PROTECT study. Overall, compared to the anticoagulation medical therapy, the LAAC device showed a statistically significantly lower risk of the composite primary outcome, CV death, all-cause deaths, and nonprocedural relevant bleeding. When compared to medical management. Considering the results of this meta-analysis, LAAC has comparable efficacy in preventing strokes and can be even greater with a longer follow-up period.

## Data Availability

The data that constitute findings of the study are available in the study itself.

## Research registration unique identifying number (UIN)

PROSPERO CRD42024538521.

## Type of study

Systematic Review, Meta-Analysis, and Meta-Regression.
